# Simulated gastric hydrolysis and developmental toxicity of dimethyltin bis(2-ethylhexylthioglycolate) in rats

**DOI:** 10.3389/ftox.2023.1122323

**Published:** 2023-02-22

**Authors:** Dominik Kirf, Richard Costlow, Hans Nasshan, Peter Frenkel, Donna Mondimore

**Affiliations:** ^1^ Chemservice S.A., Mertert, Luxembourg; ^2^ Richard Costlow Consulting LLC, Lansdale, PA, United States; ^3^ Galata Chemicals GmbH, Lampertheim, Germany; ^4^ Galata Chemicals LLC, Jersey City, NJ, United States; ^5^ PMC Group Inc., Mount Laurel, NJ, United States

**Keywords:** teratology, rat, dimethyltin bis(2-ethylhexylthioglycolate), read-across, gastric hydrolysis, CAS 57583-35-4

## Abstract

Dimethyltin dichloride is used as the putative toxophore for dimethyltin bis-alkylthio esters in a read-across approach. Recent chemical and toxicological investigations challenges this read across as data on dioctyltin bis(2-ethylhexyl thioglycolate) and dibutyltin bis(2-ethylhexyl thioglycolate) showed the dialkyltin thioglycolates do not generate dialkyltin dichloride. Results obtained by ^119^Sn-NMR spectroscopy demonstrated that dimethyltin bis(2-ethylhexyl thioglycolate), the smallest commercially manufactured dialkyltin thioester molecule of this kind, hydrolyzed to dimethyltin chloro-(2-ethylhexyl) thioglycolate under simulated gastric conditions. These studies did not detect dimethyltin dichloride. Dimethyltin bis(2-ethylhexyl thioglycolate) was administered orally to timed-pregnant Wistar-Han rats in an Arachis oil vehicle at 5, 10, and 25 mg/kg/day [Gestation Day 6 (GD6) through GD20] with no maternal deaths observed. At 25 mg/kg/day treatment statistically significant reductions occurred in feed consumption (−9%), maternal body weight (−2.4%) and adjusted maternal weight gain (−68%). There were no adverse gestational findings. Maternal thymus weight was significantly reduced in rats at 25 mg/kg in the absence of changes in hormone levels of T3, T4 or TSH. There were no effects on fetal growth, no dose-dependent pattern of external, visceral, or skeletal malformations and no toxicologically relevant increase in anatomical variations at any dose group. Based on the obtained experimental data it is concluded that dimethyltin bis(2-ethylhexyl thioglycolate) forms dimethyltin chloro-(2-ethylhexyl thioglycolate), not dimethyltin dichloride, in the stomach environment at pH 1.2, and dimethyltin bis(2-ethylhexyl thioglycolate) was not teratogenic nor fetotoxic in rats. The maternal NOAEL was 10 mg/kg/day, and the developmental NOAEL was 25 mg/kg/day, the high dose. The maternal LOAEL was 25 mg/kg/day based on decreased food consumption, lower adjusted mean body weight gain and reduced maternal thymus weight.

## Introduction

Organotin compounds have a tetragonal structure with one carbon–tin bond at a minimum, and are classified as mono-, di-, tri- and tetra-alkyltins, depending on the number of alkyl groups (Dopp et al., 2007). Organotin compounds have broad reaching applications as pesticides, stabilizers for polyvinyl chloride (PVC) piping, biocides, catalyst and antifouling agents. The alkylated tin (IV) derivatives dimethyltin dichloride (DMTC), dibutyltin dichloride (DBTC), monobutyltin trichloride (MBTC), and monomethyltin trichloride (MMTC), are commonly utilized as raw materials for the production of effective stabilizers for PVC piping (DeWitt et al., 2007). Additionally, organotin compounds have emerged as potential anti-tumour, anti-inflammatory, and antimalarial agents as well as antimicrobial agents due to enhanced biological activity post ligand-receptor binding (Syed Annuar et al., 2021). Indeed, organotin (IV) compounds when applied as anti-cancer metallodrugs, have efficacy comparable to the platinum-based cisplatin (Attanzio et al., 2020). DMTC is associated with neurotoxicity, trimethyltin chloride and dioctyltin dichloride (DOTC) are associated with thymic toxicity which is dose dependent and reversible (Menke et al., 2012). Under the Globally Harmonized System of Classification and Labelling of Chemicals (GHS) and the legal framework for classification and labelling in the EU (CLP Regulation) DMTC is classified as Reprotoxicant of Category 2 (H361d) as it induced developmental toxicity in experimental fertilized animals (ECHA, 2012). Studies demonstrated DMTC reduced thymus weight and foetal weight in pregnant rats at a LOAEL of 15 of mg/kg bw/day (Noda, 2001) with the development of cleft palates promoting classification as a developmental toxicant. As a result of application of a read across approach other alkyltin derivates were classified as developmental toxicants based on a hypothesis that DMTC was a common putative metabolite and the proximate toxophore for the thymic toxicity and the developmental effects. The read across and the categorical grouping of compounds is a recognized approach for the safety and risk assessment of chemicals at international level (Beker van Woudenberg et al., 2013). Read across is not without limitations in regulatory toxicology, standardization and a lack of comparative animal testing for chemical compounds of similar structure are challenges evident (Chesnut et al., 2018). Whilst a number of resources including published literature, chemical databases, and computational resources are available for supporting the determination of chemical similarity, the biological activity of compounds is still of great importance for the read across approach (Ball et al., 2016). Thus, non-testing methods, should be combined with an *in vitro* or *in vivo* test battery to more accurately determine toxicity profiles.

The read across hypothesis that DMTC was a putative metabolite of certain alkyltin esters and the proximate toxophore for the thymic and developmental toxicity was primarily based on the following investigations. Simulated gastric hydrolysis data (Yoder, 2000a; Yoder, 2000b; ORTEP, 2000; Schilt and Zondervan-van den Beuken, 2004; Ghobrial et al., 2019) suggested that several alkyltin esters were hydrolyzed to their respective alkyltin chlorides in acidic media. Existing reviews by regulatory bodies (Organization for Economic Cooperation and Development, 2006; KEMI, 2018) utilized the assumption that DMTC was the toxophore for certain methyltin compounds.

Simulated gastric hydrolysis conducted on dioctyltin bis(2- ethylhexyl thioglycolate) (DOTE) and dibutyltin bis(2-ethylhexyl thioglycolate) (DBTE) however, has demonstrated that hydrolysis to the dichloride toxophore is not standard for all dialkyltin derivatives. More specifically, Costlow et al., 2017, detected no toxophore DOTC formation following gastric stimulation conditions of DOTE with no developmental toxicity observed in exposed rabbits (Costlow et al., 2017). Additionally, for DBTE, no dibutyl dichloride toxophore was detected following gastric simulation and orally administered DBTE produced no maternal deaths, no treatment-related statistically significant reductions in feed consumption or maternal body weight and no adverse gestational outcomes in rats (Costlow et al., 2021). Therefore, such experimental investigation was also warranted for the organotin compound dimethyltin bis(2-ethylhexyl thioglycolate) (DMTE) to verify its suitable hazard classification based on empirical data on the substance. The investigations described herein included a simulated gastric hydrolysis of DMTE with the use of ^119^Sn-NMR spectroscopy to determine if the dichloride toxophore if formed from DMTE. The developmental toxicity of DMTE (98.0% purity) was also investigated, where results highlight that developmental toxicity as a nefarious outcome is not guaranteed for this compound.

## Materials and methods

The study was conducted at Charles River Laboratories Den Bosch B.V. (Study No. 20266382) following OECD Guideline 414 (Prenatal Developmental Toxicity Study, June 2018) and in accordance with the OECD Principles of Good Laboratory Practice (GLP). The test material for all studies was dimethyltin bis(2-ethylhexylthioglycolate) (DMTE; CAS No. 57583-35-4; IUPAC: 2-ethylhexyl 4,4-dmethyl-10-ethyl-7-oxo-8-oxa-3,5-dithia-4-stannatetradecan-1-oate) supplied by Galata Chemicals, GmbH, Lampertheim, Germany: for hydrolysis, Batch AK114/089/01, >90% purity]; for toxicology, 98% purity, supplied by PMC Group. Food grade arachis oil [Batch AR03 Fagron, Capelle aan den IJssel, Netherlands] was used as a vehicle. Fresh formulations were prepared daily for administering DMTE.

Hydrolytic stability of DMTE was conducted in accordance with OECD Guideline Number 111 measured at pH 4.0, 7.0, 9.0 at 50°C for 120 h. Additional measurements were made at pH 1.2°C and 37°C at different time intervals up to 72 h. The experiments were run in duplicate. The solutions for each tested pH were obtained from VWR International GmbH. The pH 1.2 medium was a 0.1 M aqueous HCl solution. NMR Instrument: Bruker Avance 200; sample preparation: 370 μl/330 μl toluene-d8 (10 mg/ml CrAcAc).

Sample preparation was as follows: 1 g (1.8 mmol) of pre-heated DMTE was added to 100 ml pH 1.2 medium heated to 37°C with stirring. At specified times the reaction mixtures were extracted with hexane. After removal of the solvent, remaining residues were analyzed by ^119^Sn-NMR spectroscopy.

The developmental studies were performed in a facility accredited by the American Association for Accreditation of Laboratory Animal Care (AAALAC), complied with the Principles of Good Laboratory Practices, and followed Organization for Economic Cooperation and Development (OECD) Test Guideline Number 414. Animal welfare at the facility complied with procedures approved by the Central Authority for Scientific Procedures on Animals (CCD) as required by the Dutch Act on Animal Experimentation (December 2014).

Time-mated rats (Wistar Han strain) were received on Day zero or Day 1 post-coitum from Charles River Deutschland, Sulzfeld, Germany. They were provided 5 days of acclimation and were 10–14 weeks of age at the initiation of dosing. Rats were housed in a controlled environment. Conditions were monitored to provide >10 air changes per hour, temperature 18–24 C, relative humidity 40%–70%, 12 h of light and 12 h of darkness. Polycarbonate cages (Makrolon type MIII 18 cm high) with sterilized wood fiber bedding material were used. Rats were supplied aspen wood sticks as environmental enrichment and were fed *ad libitum* with SM R/M-Z from SSNIFF® Spezialdiäten GmbH, Soest, Germany in pellet form throughout the experimental period.

Municipal tap water was provided *ad libitum* throughout the experimental period in water bottles.

Gestation Day 0 (GD0) was the day of successful mating. Presumed-pregnant females were housed individually in polycarbonate cages. Female rats were randomly assigned to each of 4 groups, 22 presumed-pregnant females per group. Dosing solutions were prepared and administered daily by oral gavage on GD6 through GD20.

Fetuses were removed on GD21 by caesarean section. Live fetuses were allocated for subsequent evaluations independent of sex. After being euthanized, half the fetuses received visceral examinations and half received skeletal examinations for cartilage and bone. Approximately one-half of live fetuses from each litter were decapitated; the heads were fixed in Bouin’s solution and subjected to free-hand serial sectioning, also known as the Wilson technique. Visceral examinations were performed using micro-dissection, also known as the Staples technique. Fetuses were stained with Alcian blue for cartilage, and with Alizarin red S for calcified structures.

Statistical analyses were conducted using commercially available software and appropriate datasets. Levene’s test was used to assess the homogeneity of group variances. Other tests: one-way ANOVA F-test or Kruskal Wallis, as appropriate with Dunnett’s or Dunn’s post-test, respectively, for parametric data including maternal body weight, weight change, corrected body weight, thymus weight, uterus weight, feed consumption, fetal weight, fetal C-R length. For non-parametric data: Kruskal–Wallis test followed by Mann-Whiney test including number of corpora lutea, number of implantations, litter size, number of live fetuses, percentage of early and late resorptions, percentage of visceral or skeletal variations or malformations for each litter, percentage of individual variations or malformations for each litter.

Dose solutions were chemically analyzed during the study and were within 15% of the nominal concentrations. DMTE doses were 5, 10 and 25 mg/kg bw/day in a dose volume of 5 ml/kg. The Control group received Arachis oil vehicle at the same dose volume.

## Results

### Hydrolysis in simulated gastric Milieu

**FIGURE 1:**
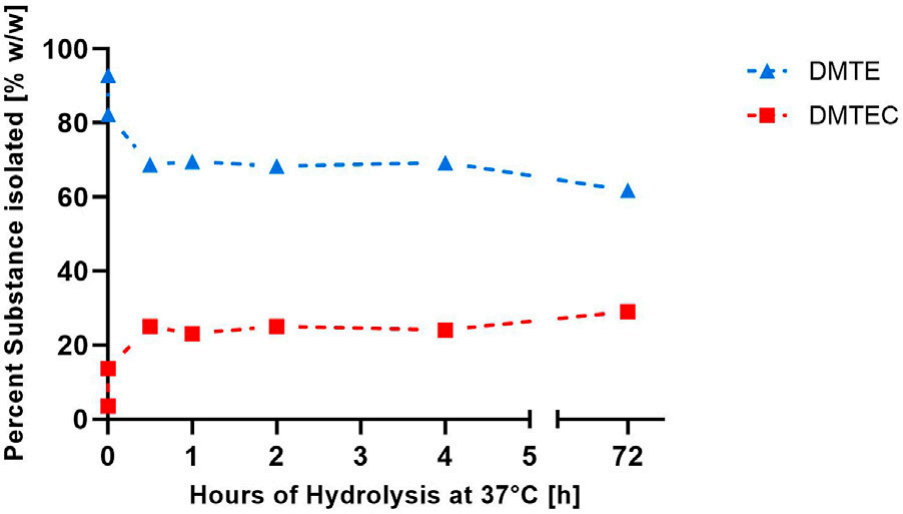
Percent weight/weight of DMTE and DMTEC isolated from the simulated gastric media (pH 1.2°C and 37°C) from zero to 72 h.

The results in Tables 1, 2 and Figure 1 were obtained from the experimental gastric simulation system. They demonstrate that DMTE equilibrated rapidly under the study conditions of pH 1.2 at 37°C. DMTC was not detected. The initial concentration of DMTE was 92.9%. Concentration decreased to 82.3% within seconds and was 68.7% at the 30-min point. DMTE equilibrated in the narrow concentration range of 68%–70% up to the 4-h point and declined only slightly thereafter to 61.8% at 72 h. The mono-chloro-thioester, dimethyltin chloro-(2-ethylhexyl thioglycolate) (DMTEC) concentration, which was initially present only as an impurity at 3.5%, increased to 13.7% within seconds, reached 25.0% at the 30-min point, and fluctuated in the narrow range of 23%–25% up to the 4-h point. A slight increase to 29.0% was observed at 72 h.

**TABLE 1:** Hydrolysis of DMTE in gastric simulation media at pH 1.2°C and 37°C for 72 h.

Time [h]	DMTE [%w/w]	DMTEC [%w/w]
0.0	92.9	3.5
0.001	82.3	13.7
0.5	68.7	25.0
1.0	69.5	23.0
2.0	68.3	25.0
4.0	69.2	24.0
72.0	61.8	29.0

**TABLE 2:** Maternal thymus and thyroid data of rats exposed to DMTE.

DMTE Dose [mg/kg/day]	Thymus weight [g] (n)	Thyroid weight [g] (n)	T3 [ng/ml] (n)	TSH [mU/ml] (n)	T4 [ng/ml] (n)
0	0.1927 ± 0.0476 (21)	0.01340 ± 0.00262 (21)	0.368 ± 0.101 (20)	0.4264 ± 0.3230 (21)	21.39 ± 6.11 (20)
5	0.2158 ± 0.0486 (21)	0.01436 ± 0.00263 (21)	0.330 ± 0.065 (21)	0.3275 ± 0.1422 (21)	21.12 ± 4.64 (21)
10	0.1828 ± 0.0428 (21)	0.01303 ± 0.00361 (21)	0.334 ± 0.132 (22)	0.4800 ± 0.2682 (22)	18.13 ± 6.40 (22)
25	0.1608* ± 0.0332 (22)	0.01343 ± 0.00234 (22)	0.338 ± 0.092 (22)	0.5285 ± 0.2725 (22)	19.68 ± 4.94 (22)

Mean and Standard Deviation of maternal thymus weights [g], maternal thyroid weight, and maternal thyroid hormones of rats exposed to DMTE, at the indicated doses are presented.

(*) indicates a statistically significant difference (*p* < 0.05).

Bold value represents the most important as statistical significance was reached.

A mono-methyltin component, monomethyltin tris(2-ethylhexylthioglycolate) (MMTE), initially present in the DMTE sample at ∼5%, did not affect the outcome with DMTE.

**FIGURE 2:**
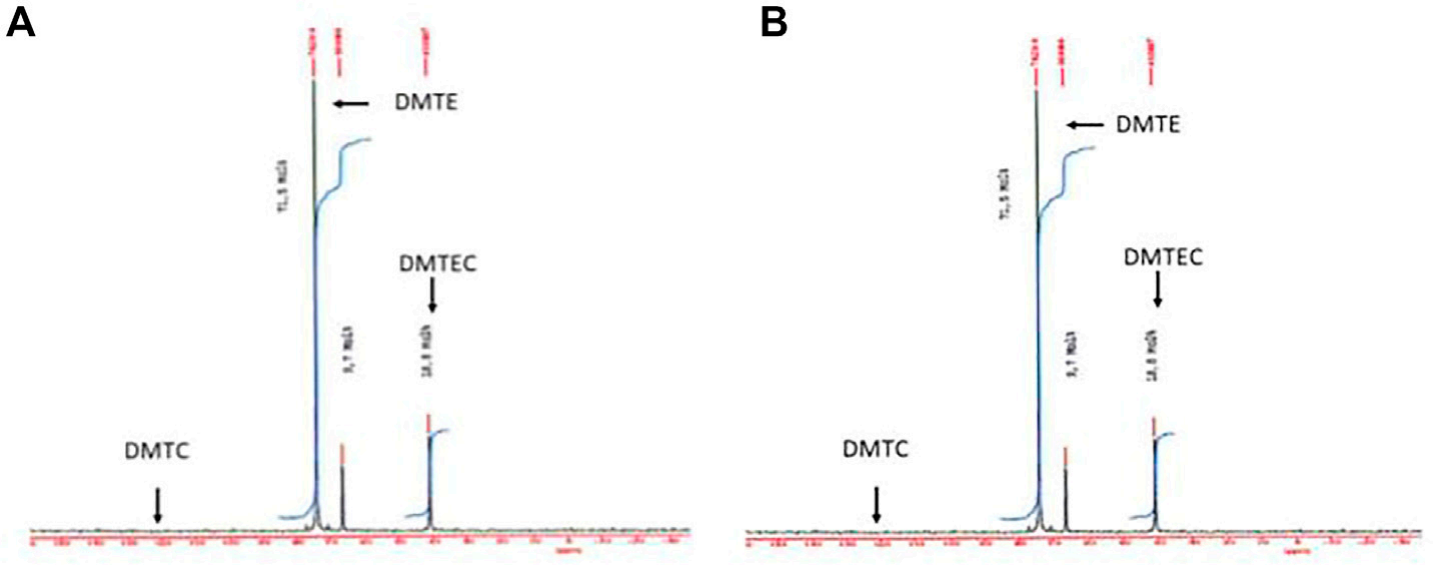
^119^Sn-NMR spectra of the material isolated from the simulated gastric hydrolysis are presented. **(A)** DMTE after 30 s in gastric media at pH 1.2 and 37°C; **(B)** DMTE after 4 h in gastric media at pH 1.2 and 37°C.

In Figure 2 the ^119^Sn-NMR spectra of the hydrolysate showed about 65% decrease of the signal intensity at 74 ppm attributed to DMTE, whereas the DMTEC signal at 41 ppm increased by the same amount. A signal for DMTC would have been expected at 131 ppm and it was not detected there (the detection limit for DMTC with the instrument under the conditions of the study was 0.5%).

The significant shift of the ^119^Sn-NMR signal from 74 ppm to 41 ppm is attributed to the formation of DMTEC. It represents a molecular structure where the central tin atom has a higher coordination resulting in a high field shift. This type of coordination must enable steric hindrance of the molecular structure in general and electron-stabilization of the tin atom in particular.

A comparable behavior was observed with the MMTE component (67 ppm). After 72 h a new signal appeared at −13 ppm with an intensity of 1.3%. The new peak is attributed to the formation of mono-methyltin chloro-bis(2- ethylhexylthioglycolate) (MMTE2C).

Based on the experimental results, the mechanism of DMTE hydrolysis can be expressed as shown in Eq. 1.

It is concluded that DMTEC is the only tin-containing metabolite of DMTE that was formed under conditions of the simulated mammalian gastric environment. No DMTC was detected under the conditions of the study.

### Maternal and developmental outcomes

The dose levels administered to rats in the main study were 0, 5, 10, and 25 mg/kg bw/day of DMTE. These doses were based on data from an identically designed range-finding study at doses of 0, 25, and 50 mg/kg bw/day in which clear treatment-related adverse effects on maternal weight occurred at 25 mg/kg bw/day, and maternal rats required euthanasia in extremis at 50 mg/kg bw/day (4/6).

There were no maternal mortalities at any dose in the main study. Maternal rats in the 25 mg/kg group of the main study showed treatment-related signs of toxicity, approximately 10% decreased feed consumption compared to controls, and lower unadjusted maternal body weight at the end of gestation. These effects were deemed treatment-related though not statistically significant.

Data in Table 3 indicate that mean gravid uterus weight of treated females at all doses was in the same range as controls. Mean maternal body weight gain adjusted for gravid uterus weight was reduced at 25 mg/kg/day to 32% of the controls, and it was reduced at 10 mg/kg/day to 68% of controls. Both mean values were below the lower end of available Historical Control Data.

**TABLE 3:** Maternal reproductive success and fetal development of rats exposed to DMTE.

DMTE Dose [mg/kg bw/day]	0	5	10	25
Number Presumed Pregnant	22	22	22	22
Confirmed pregnant	22	21	22	22
Mean Terminal Body weight [g]	307.0	315.0	307.9	299.8
Body weight gain [g] GD21-GD6	84.5	89.5	83.5	74.1
Mean Gravid uterus weight [g]	68.12	72.75	73.63	68.94
Corrected weight gain^a^ [g]	16.40	16.72	11.32	5.20*
Corrected weight gain [% of Control]	n/a	102%	68%	32%
Mean Corpora Lutea	11.5	11.7	12.2	11.3
Mean Implantation Sites	10.6	10.5	11.2	10.2
Live Fetuses/Litter	10.4	10.3	10.8	10.0
Non-viable Fetuses (Litters)	0 (0)	0 (0)	0 (0)	0 (0)
Pre-implantation Losses^b^	7.00%	9.31%	6.86%	9.06%
Post-implantation Losses	1.90%	2.17%	3.82%	2.92%
Mean Fetal Body Weight [g] (±SD)	4.949 (0.534)	5.358* (0.282)	5.132 (0.348)	5.148 (0.430)
Mean Fetal Anogenital Distance [mm], males (±SD)	2.60 (0.22)	2.74 (0.28)	2.69 (0.31)	2.72 (0.27)
Mean Fetal Anogenital Distance [mm], females (±SD)	1.10 (0.17)	1.22 (0.24)	1.17 (0.21)	1.16 (0.14)
Sex Ratio [M/F]	1.17	1.13	1.01	0.79
Fetuses examined [Litters]	219 [21]	216 [21]	226 [21]	219 [22]
Fetuses examined externally	219	216	226	219
Fetuses examined viscerally	110	107	112	110
Fetuses examined skeletally	109	109	114	109
No. of External Malformations [Litters]	0 [0]	1 [1]	0 [0]	0 [0]
No. of Visceral Malformations [Litters]	1 [1]	0 [0]	0 [0]	0 [0]
No. of Skeletal Malformations [Litters]	1 [1]	1 [1]	0 [0]	1 [1]
No. of External Variations [Litters]	1 [1]	0 [0]	2 [2]	0 [0]
No. of Visceral Variations [Litters]	4 [4]	5 [3]	3 [5]	6 [3]
No. of Skeletal Variations [Litters]	73 [20]	83 [21]	75 [21]	88 [22]
External Malformations				
*Short tail [Litters]*	0 [0]	0 [0]	1 [1]	0 [0]
Visceral Malformations				
*Brain ventricles dilated (bilateral) [Litters]*	0 [0]	1 [1]	0 [0]	0 [0]
*Eye (right) absent [Litters]*	0 [0]	1 [1]	0 [0]	0 [0]
*Eye (left) small [Litters]*	0 [0]	1 [1]	0 [0]	0 [0]
*Situs inversus [litters]*	1 [1]	0 [0]	0 [0]	0 [0]
Skeletal Malformations				
*Humerus bent (right) [Litters]*	0 [0]	1 [1]	0 [0]	0 [0]
*Supernumerary sternebra [Litters]*	0 [0]	0 [0]	0 [0]	1 [1]
*Supernumerary vertebra (cervical) [Litters]*	0 [0]	0 [0]	0 [0]	1 [1]
*Supernumerary vertebra (lumbar) [Litters]*	0 [0]	0 [0]	0 [0]	1 [1]
Visceral Variations				
*Renal papilla absent (unilateral left) [Litters]*	0 [0]	1 [1]	0 [0]	0 [0]
*Renal papilla absent (unilateral right) [Litters]*	0 [0]	0 [0]	0 [0]	2 [1]
*Supernumerary liver lobe (left medial) [Litters]*	5 [5]	6 [4]	8 [7]	6 [4]
*Supernumerary liver lobe (right medial) [Litters]*	2 [2]	2 [2]	4 [3]	4 [4]
*Pale-colored spleen [Litters]*	1 [1]	0 [0]	0 [0]	0 [0]
*Dilated ureter (bilateral) [Litters]*	0 [0]	0 [0]	0 [0]	1 [1]
*Dilated ureter (right) [Litters]*	0 [0]	0 [0]	0 [0]	1 [1]
*Convoluted ureter (left) [Litters]*	0 [0]	2 [2]	0 [0]	0 [0]
*Convoluted ureter (right) [Litters]*	0 [0]	0 [0]	0 [0]	1 [1]
Skeletal Variations				
*Frontal skull plate(s) poorly ossified [Litters]*	1 [1]	0 [0]	1 [1]	2 [1]
*Interparietal skull plate poorly ossified [Litters]*	2 [2]	2 [2]	4 [3]	2 [1]
*Parietal skull plate(bilateral) poorly ossified [Litters]*	2 [2]	0 [0]	0 [0]	1 [1]
*Parietal skull plate(left) poorly ossified [Litters]*	0 [0]	0 [0]	1 [1]	0 [0]
*Parietal skull plate(right) poorly ossified [Litters]*	1 [1]	0 [0]	0 [0]	2 [1]
*Sternebra (>1) misaligned [Litters]*	3 [3]	1 [1]	1 [1]	1 [1]
*Sternebra (>1) poorly ossified [Litters]*	1 [1]	1 [1]	1 [1]	1 [1]
*Sternebra (>1) not ossified [Litters]*	6 [2]	0 [0]	1 [1]	0 [0]
*Costal cartilages (>1) fused [Litters]*	0 [0]	0 [0]	0 [0]	1 [1]
*Wavy rib(s) [Litters]*	30 [14]	24 [11]	18 [8]	4 [4]*
*Supernumerary rib (cervical and short) (>1) [Litters]*	9 [7]	5 [3]	6 [5]	8 [6]
*Supernumerary rib (thoracolumbar and full) (>1) [Litters]*	6 [4]	10 [6]	4 [4]	8 [6]
*Supernumerary rib (thoracolumbar and short) (>1) [Litters]*	49 [17]	64 [21]	61 [21]	78 [22]*
*Scapula bent (right) [Litters]*	0 [0]	1 [1]	1 [1]	0 [0]
*Metacarpals (>1) unossified [Litters]*	7 [2]	0 [0]	0 [0]	0 [0]
*Ilium misaligned (bilateral) [Litters]*	3 [2]	6 [4]	4 [3]	3 [3]
*Thoracic centra (>1) poorly ossified [Litters]*	0 [0]	3 [3]	0 [0]	1 [1]

^a^ Corrected body weight gain is (GD21 minus GD6 weight) minus GUW; where GUW, gravid uterine weight.

^b^ Study utilized timed-pregnant females; dosing began after implantation so pre-implantation losses cannot be related to treatment.

*Statistically significant (*p* < 0.05).

Bold value represents the most important as statistical significance was reached.

Lower thymus weights were recorded for dams only in the 25 mg/kg bw/day group at approximately 15% of the control, though this difference was not statistically significant. Thyroid weight, macroscopic appearance, and levels of TSH were within historical control values at all doses. No thyroid histopathology was remarkable; the prevalence, severity, and histologic characterization were deemed incidental tissue alterations.

The maternal reproductive parameters of gravid uterine weight, resorptions per litter, live and dead fetuses, and fetal sex ratio were all within historical control limits for all treated groups. The calculated incidence of post-implantation loss was also within historical control limits for all treated groups (Table 3). It was taken into consideration that calculation of pre-implantation loss is not relevant given that dose administration began after the implantation.

Fetal evaluations at term indicated no treatment-related difference in mean litter size, sex ratio, mean fetal body weight or mean fetal crown-to-rump length. The statistically significant increase in mean fetal weight in the 5 mg/kg/day group was deemed incidental and a normal physiological variation.

Observations of external, visceral and skeletal malformations were limited to single litters and single fetuses within those litters. These findings are morphologically not related and/or show no dose-dependency and were thus considered as incidental findings.

The observed soft tissue variations included supernumerary liver lobes, convoluted and/or dilatation ureter, and missing renal papilla. The distribution of these findings was not treatment related. Similarly, the small number of skeletal variations were without a demonstrable dose-related incidence. There was a statistically significant reduction in the number of fetuses with wavy ribs, and a statistically significant increase in the number of fetuses with supernumerary thoracolumbar short ribs at 25 mg/kg/day. However, wavy ribs are transient variations, and a (incidental) lower incidence is not toxicologically relevant. Short thoracolumbar ribs will become part of the transverse process of the vertebrae (Hofmann et al., 2016). Therefore, this finding should be considered as transient variation and is considered to be not toxicologically relevant.

Overall, there was no increased incidence of external, visceral or skeletal malformations or variations associated with treatment with DMTE.

## Discussion

The results of this study describe the findings of simulated gastric hydrolysis as well as developmental toxicity studies of three substances, DMTE, DBTE and DOTE, that are widely used as heat stabilizer components for compounding of PVC, its co-polymers and CPVC. These data substantively challenge the validity of applying the read-across from the results of mammalian studies with the respective dialkyltin dichlorides, DMTC, DBTC and DOTC, to derive the hazard classifications for the corresponding dialkyltin dithioglycolates. Prior to the availability of these ^119^Sn-NMR data, use of read-across for toxicological hazard classification in these cases was uniquely attributed to the former analytical method and sensitive to the shortcomings of underlying assumptions or to artifacts not obvious when structural similarity alone was considered.

Previous hydrolysis studies of DMTE and other dialkyltin bis-2-ethylhexylthioglycolates (Bautista and Herzig, 2000; Yoder, 2000b; Gillard-Factor and Yoder, 2000) used Gas Chromatography equipped with Flame Ionization Detection (FID) to measure 2-ethylhexyl thioglycolate (EHTG) and 2-ethylhexanol (EH), hydrolysis breakdown products of DMTE. This analytical method did not allow for direct determination of the tin moieties. Deficiencies of this method were attributed to interference between the DMTE derived EHTG and EH peaks, which had almost the same retention time. Additionally, both EHTG and EH peaks were asymmetrical (“tailing”), inflating the sum of the peak areas. Thioglycolic acid (TGA), another breakdown product of EHTG, was not accounted for, further exaggerating the total peak. These factors likely led to the incorrect inference that DMTE hydrolyzed to DMTC.

Toxicological read-across from DMTC to DMTE, based on their structural similarity was supported solely by analytical data from the simulated gastric hydrolysis study proven now to have methodological artifacts. The experimental results presented here show the simulated gastric metabolism of DMTE, in failing to hydrolyze *in vitro* to DMTC, is entirely consistent with results from similar studies with DOTE (Nasshan, 2015b; Costlow et al., 2017) and DBTE (Nasshan, 2015a; Costlow et al., 2021). Results of the developmental toxicity study with DMTE further challenge the applicability of the dialkyltin dichloride to dialkyltin bis-thioglycolate read-across in the DMTE case.

These data, investigating three dialkyltin bis-thioglycolate substances, showed the presence of both the EHTG ligand and the chlorine atom bound to the dialkyltin center of the thermodynamically stable dialkyltin chloro-(2-ethyhexyl thioglycolate), a structure of which precludes its further metabolic hydrochlorination and formation of dialkyltin dichlorides (Schiller, 2022). This likely has profound significance for their non-reprotoxic characteristics.

The DMTE data also adds to the significant body of evidence which indicates dithioglycolate-dialkyltin substances are not reproductive toxicants. The use of data from studies with dichloro-dialkyltin substances which are reproductive toxicants to read-across a toxicological classification to a dithioglycolate-dialkyltin substance is questionable. The findings described herein, suggest that classification of DMTE as a reproductive toxicant as derived from a read-across approach, warrants additionally investigative studies.

Results of the experimental developmental toxicity study with DMTE demonstrate that DMTE is not teratogenic nor is it a developmental toxicant in rats. When compared to the WHO GHS classification criteria, these data in conjunction with the implications of the simulated gastric hydrolysis substantiate a conclusion that DMTE should not prematurely be classified as a developmental toxicant.

## Data Availability

The original contributions presented in the study are included in the article/Supplementary Material, further inquiries can be directed to the corresponding author.

## References

[B1] Attanzio A. D'Agostino S. Busa R. Frazzitta A. Rubino S. Girasolo M. A. (2020). Cytotoxic activity of organotin(IV) derivatives with triazolopyrimidine containing exocyclic oxygen atoms. Molecules 25 (4), 859. 10.3390/molecules25040859 32075253 PMC7070731

[B2] Ball N. Cronin M. T. Shen J. Blackburn K. Booth E. D. Bouhifd M. (2016). Toward Good read-across Practice (GRAP) guidance. ALTEX 33 (2), 149–166. 10.14573/altex.1601251 26863606 PMC5581000

[B3] Bautista A. Herzig L. (2000). Simulated gastric hydrolysis of butyltin and octyltin mercaptides. Elf Atochem North America Report. report dated 2000-05-23.

[B4] Beker van Woudenberg A. Wolterbeek A. Te Brake L. Snel C. Menke A. Rubingh C. (2013). A category approach to predicting the developmental (neuro) toxicity of organotin compounds: The value of the zebrafish (*Danio rerio*) embryotoxicity test (ZET). Reprod. Toxicol. 41, 35–44. 10.1016/j.reprotox.2013.06.067 23796951

[B5] Chesnut M. Yamada T. Adams T. Knight D. Kleinstreuer N. Kass G. (2018). Regulatory acceptance of read-across. ALTEX 35 (3), 413–419. 10.14573/altex.1805081 30008009

[B6] Costlow R. D. Nasshan H. Frenkel P. Salsbury J. (2021). Simulated gastric hydrolysis and developmental toxicity of dibutyltin bis(2-ethylhexyl thioglycolate) in rats. J. Appl. Toxicol. 41 (11), 1794–1802. 10.1002/jat.4162 33774828

[B7] Costlow R. D. Nasshan H. Frenkel P. (2017). Simulated gastric hydrolysis and developmental toxicity of dioctyltin bis(2-Ethylhexylthioglycolate) [DOTE] in rabbits and mice. Regul. Toxicol. Pharmacol. 87, 23–29. 10.1016/j.yrtph.2017.03.026 28456493

[B8] DeWitt J. C. Copeland C. B. Luebke R. W. (2007). Immune function is not impaired in Sprague-Dawley rats exposed to dimethyltin dichloride (DMTC) during development or adulthood. Toxicology 232 (3), 303–310. 10.1016/j.tox.2007.01.017 17321662

[B9] Dopp E. Hartmann L. M. von Recklinghausen U. Florea A. M. Rabieh S. Shokouhi B. (2007). The cyto- and genotoxicity of organotin compounds is dependent on the cellular uptake capability. Toxicology 232 (3), 226–234. 10.1016/j.tox.2007.01.014 17316952

[B10] European Chemical Agency [ECHA] (2012). CLH report proposal for harmonised classification and labelling based on regulation (EC) No 1272/2008. (CLP Regulation), Annex VI, Part 2. Available at: https://echa.europa.eu/documents/10162/78f994d3-b11b-fc41-0d31-8d62e369ce4a .

[B11] Ghobrial M. Stocker E. Hölzl C. Mihovilovic M. Stanetty C. (2019). Conversion of organotin compounds in the gastric environment. Austrian Environment Agency [umweltbundesamt] Reports Band-0709, Available at: https://www.umweltbundesamt.at/studien-reports/publikationsdetail?pub_id=2287&cHash=c44ff4a978894aec69058b10fa3e7b69 .

[B12] Gillard-Factor R. Yoder R. (2000). MS study of the hydrolysis of various organotins under simulated gastric conditions. Elf Atochem North America. report dated 2000-05-23.

[B13] Hofmann T. Buesen R. Schneider S. van Ravenzwaay B. (2016). Postnatal fate of prenatal-induced fetal alterations in laboratory animals. Reprod. Toxicol. 61, 177–185. 10.1016/j.reprotox.2016.04.010 27094378

[B14] Menke A. Wolterbeek A. Snel C. Bruijntjes J. de Groot D. van Oostrum L. (2012). Potentially increased sensitivity of pregnant and lactating female rats to immunotoxic agents. Toxicol. Pathol. 40 (2), 255–260. 10.1177/0192623311428476 22089841

[B15] Nasshan H. (2015b). Galata chemicals GmbH, chemiestrasse 22, 68623 Lampertheim, Germany. Report dated 2015-04-05.DOTE - Hydrolysis as a Function of pH.

[B16] Nasshan H. (2015a) Lampertheim, Germany. Report dated 2015-01-27.Dibutyltin bis(2-ethylhexylthioglycolate) [DBTE] *in vitro* metabolism study. Chemiestrasse. 22. Galata Chemicals GmbH, 68623.

[B17] Noda T. (2001). Maternal and fetal toxicity of dimethyltin in rats. J. Health Sci. 47 (6), 544–551. 10.1248/jhs.47.544

[B18] Organization for Economic Cooperation and Development [Oecd] (2006). SIDS initial assessment profile, from information assessment meeting. [SIAM] Number 23, 17-20 October 2006. Available at: https://hpvchemicals.oecd.org/UI/SIDS_Details.aspx?key=b8a494fe-ca60-47eb-abcb-188c0bd7d41e&idx=0 .

[B19] Organotin Environmental Program Stabilizer Task Force [ORTEP STF] (2000). Summary report on the simulated gastric hydrolysis of tin mercaptide stabilizers.

[B20] Schiller M. (2022). PVC additives: Performance, chemistry, developments, and sustainability. 2nd Edition. München: Carl Hanser Verlag), 27–28.

[B21] Schilt R. Zondervan-van den Beuken E. K. (2004). Dibutyltin dilaurate (CASRN 77-58-7), dibutyltin maleate (CASRN 78-04-6), dibutyltin oxide (CASRN 818-08-6), and dioctyltin oxide (CASRN 870-08-6): Simulated Gastric Hydrolysis. TNO Nutrition and Food Research. Report Number V5047.

[B22] Swedish Chemicals Agency [KEMI] (2018). Grouping of Chemical substances in the REACH and CLP regulations, 49–50. Available at: https://www.kemi.se/en/publications/pms/2018/pm-2-18-grouping-of-chemical-substances-in-the-reach-and-clp-regulations .

[B23] Syed Annuar S. N. Kamaludin N. F. Awang N. Chan K. M. (2021). Cellular basis of organotin(IV) derivatives as anticancer metallodrugs: A review. Front. Chem. 9, 657599. 10.3389/fchem.2021.657599 34368075 PMC8342812

[B24] Yoder R. (2000a). Development of a method to directly determine monobutyltin trichloride and dibutyltin dichloride under simulated gastric conditions. Elf Atochem North America. report dated 2000-05-11.

[B25] Yoder R. (2000b). Measurement of the hydrolysis of various organotin stabilizers under simulated gastric conditions. Elf Atochem North America. report dated 2000-05-15.

